# Bis{(*R*)-1-(3-amino­sulfonyl-4-meth­oxy­phen­yl)-*N*-[2-(2-eth­oxy­phen­oxy)eth­yl]propan-2-aminium} adipate tetra­hydrate

**DOI:** 10.1107/S1600536813005254

**Published:** 2013-03-09

**Authors:** Zoran Ham, Anton Meden, Marta Kasunič

**Affiliations:** a Lek Pharmaceuticals, Sandoz Development Centre Slovenia, Verovškova 57, SI-1526 Ljubljana, Slovenia; b Faculty of Chemistry and Chemical Technology, University of Ljubljana, Aškerčeva 5, SI-1000 Ljubljana, Slovenia

## Abstract

The title compound, 2C_20_H_29_N_2_O_5_S^+^·C_6_H_8_O_4_^2−^·4H_2_O, which was found to be optically active, is a relatively rare example of a chiral compound crystallizing in the triclinic crystal system. The dihedral angles between the phenyl rings of the cations are 60.03 (15) and 62.03 (16)°, while the C atoms of the anion are almost coplanar (r.m.s. deviation 0.085 Å) and all *trans* to each other. In the crystal, the components are connected by an extensive network of N—H⋯O and O—H⋯O hydrogen bonds. The sulfonamide groups link the cations into pairs *via* two N—H⋯O hydrogen bonds about the pseudo-inversion centre, leading to the formation of *R*_2_^2^(8) rings. The anions are stacked in between four cationic pairs. Pairs of water mol­ecules bridge the larger building units, forming hydrogen bonds with the remaining two O atoms of the anion.

## Related literature

(*R*)-5-(2-(2-(2-Eth­oxy­phen­oxy)ethyl­amino)­prop­yl)-2-meth­oxy­benzene­sulfonamide (generic name tamsulosin) has an α-adrenergic blocking action and possesses hypotensive activity and is used mainly for the treatment of benign prostatic hyperplasia, see: Abrams *et al.* (1995 ▶). 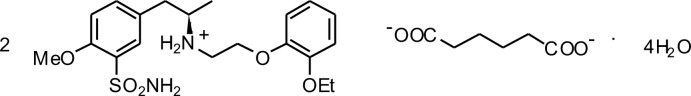


## Experimental

### 

#### Crystal data

2C_20_H_29_N_2_O_5_S^+^·C_6_H_8_O_4_^2−^·4H_2_O
*M* *_r_* = 1035.21
Triclinic, 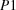

*a* = 10.4595 (2) Å
*b* = 11.9020 (3) Å
*c* = 12.6423 (3) Å
α = 69.439 (1)°
β = 70.466 (1)°
γ = 67.058 (1)°
*V* = 1320.95 (5) Å^3^
*Z* = 1
Mo *K*α radiation
μ = 0.17 mm^−1^
*T* = 293 K
0.25 × 0.25 × 0.20 mm

#### Data collection

Nonius KappaCCD diffractometer
Absorption correction: multi-scan (*DENZO-SMN*; Otwinowski & Minor, 1997 ▶) *T*_min_ = 0.933, *T*_max_ = 0.966
26272 measured reflections
10988 independent reflections
8651 reflections with *I* > 2σ(*I*)
*R*_int_ = 0.030

#### Refinement

*R*[*F* ^2^ > 2σ(*F* ^2^)] = 0.047
*wR*(*F* ^2^) = 0.143
*S* = 1.00
10988 reflections
641 parameters
7 restraints
H-atom parameters constrained
Δρ_max_ = 0.42 e Å^−3^
Δρ_min_ = −0.36 e Å^−3^
Absolute structure: Flack (1983 ▶), 4978 Friedel pairs
Flack parameter: 0.10 (8)

### 

Data collection: *COLLECT* (Nonius, 1998 ▶); cell refinement: *DENZO-SMN* (Otwinowski & Minor, 1997 ▶); data reduction: *DENZO-SMN*; program(s) used to solve structure: *SIR97* (Altomare *et al.*, 1999 ▶); program(s) used to refine structure: *SHELXL97* (Sheldrick, 2008 ▶); molecular graphics: *ORTEP-3 for Windows* (Farrugia, 2012 ▶) and *Mercury* (Macrae *et al.*, 2006 ▶); software used to prepare material for publication: *publCIF* (Westrip, 2010 ▶).

## Supplementary Material

Crystal structure: contains datablock(s) global, I. DOI: 10.1107/S1600536813005254/ld2093sup1.cif

Structure factors: contains datablock(s) I. DOI: 10.1107/S1600536813005254/ld2093Isup2.hkl

Supplementary material file. DOI: 10.1107/S1600536813005254/ld2093Isup3.cml

Additional supplementary materials:  crystallographic information; 3D view; checkCIF report

## Figures and Tables

**Table 1:** Hydrogen-bond geometry (Å, °)

*D*—H⋯*A*	*D*—H	H⋯*A*	*D*⋯*A*	*D*—H⋯*A*
N1—H1*B*⋯O21^i^	0.96	2.11	3.028 (5)	160
N11—H11*B*⋯O11^ii^	0.86	2.23	3.029 (5)	155
N2—H2*C*⋯O3*W*	0.96	1.84	2.755 (4)	160
N12—H12*B*⋯O1*W*	0.96	1.87	2.797 (4)	160
N2—H2*D*⋯O1^iii^	0.96	1.98	2.854 (4)	151
N12—H12*A*⋯O3^iv^	0.96	1.83	2.746 (4)	159
N1—H1*A*⋯O3	0.95	1.84	2.790 (5)	173
N11—H11*A*⋯O1	0.78	2.03	2.798 (6)	169
O1*W*—H1*WB*⋯O2*W* ^v^	0.95	1.80	2.746 (5)	174
O3*W*—H3*WB*⋯O4*W* ^vi^	0.96	1.82	2.747 (5)	161
O2*W*—H2*WA*⋯O2	0.95	1.75	2.690 (6)	175
O4*W*—H4*WA*⋯O4^iv^	0.89	1.77	2.658 (5)	175
O1*W*—H1*WA*⋯O24	0.90	2.33	2.899 (4)	121
O1*W*—H1*WA*⋯O25	0.90	2.08	2.963 (4)	165
O3*W*—H3*WA*⋯O14	0.92	2.39	2.920 (4)	116
O3*W*—H3*WA*⋯O15	0.92	2.03	2.935 (4)	166

Symmetry codes: (i) 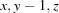; (ii) 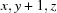; (iii) 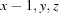; (iv) 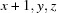; (v) 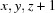; (vi) 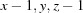.

**Fig. 1.**
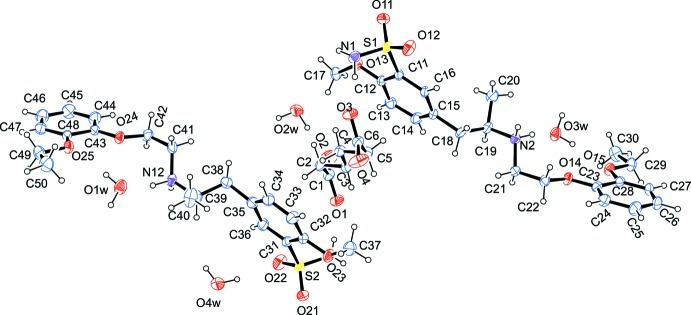
ORTEP plot of the asymmetric unit. Displacement ellipsoids are drawn at the 30% probability level and H atoms are drawn as small spheres of arbitrary radii.

**Fig. 2.**
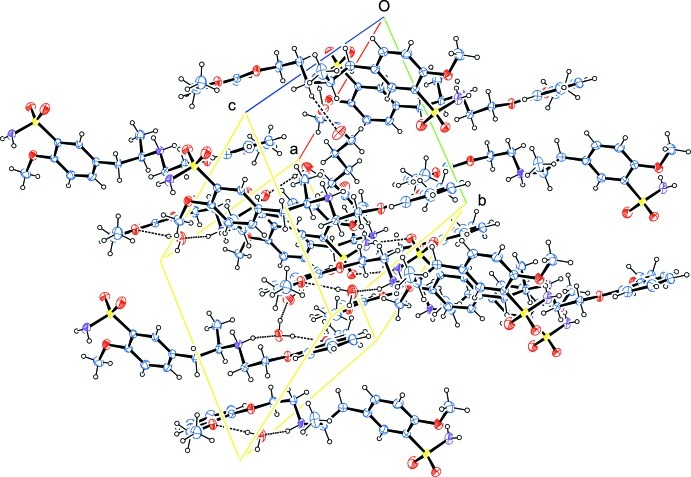
ORTEP plot of the unit-cell contents together with axis labels showing the network of hydrogen bonds. Displacement ellipsoids are drawn at the 30% probability level while and H atoms are drawn as small spheres of arbitrary radii.

**Fig. 3.**
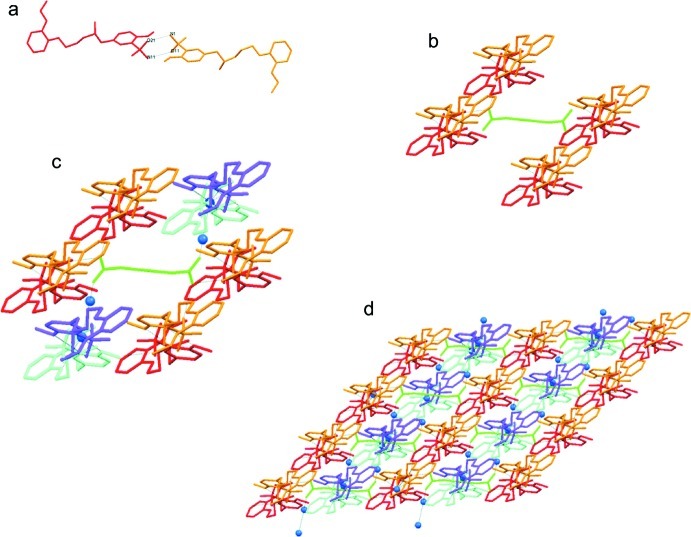
A presentation of hydrogen bonds topology starting with the formation of cationic pairs (a), larger structural segments 'A+4C' (b), revealing a role of water molecules for further connections of 'A+4C' units with additional two cationic pairs (c) leading to the final structure in which each of the cationic pairs is shared by two anions (d). Anions are drawn in green, cationic pairs in red-orange or magenta-cyan combination, and water molecules are represented by blue spheres. Hydrogen atoms have been omitted for clarity.

## supplementary crystallographic information

### Comment

(*R*)-5-(2-(2-(2-ethoxyphenoxy)ethylamino)propyl)-2-methoxybenzenesulfonamide, designated with the generic pharmaceutical name tamsulosin, has an alpha-adrenergic blocking action and possesses a hypotensive activity and is used mainly for the treatment of benign prostatic hyperplasia (BPH) (Abrams *et al.*, 1995). It has been used in the form of the hydrochloride salt of the pure *R*-enantiomer. Our attempts to prepare new salts of tamsulosin with improved solubility lead to the formation of yet unpublished tamsulosin adipate tetrahydrate (I). In this article, we report the absolute structure of (I). The asymmetric unit contains two protonated molecules of tamsulosin, adipate anion and four water molecules of solvation. All building units are connected with an extensive network of hydrogen bonds of N–H···O and O–H···O types. The former are donored by sulfonamide and amine N atoms from the cations: the shortest N···O distances and thus the strongest bonds lead towards water and anionic O atoms and are of lengths between 2.746 (4) and 2.854 (4) Å. As expected, intercationic N–H···O hydrogen bonds are somewhat weaker with N···O distances of 3.028 (5) and 3.029 (5) Å, respectively. O–H···O hydrogen bonds are donored by water molecules while the acceptors are anions, water molecules and cations, respectively. The shortest O···O distances are between water and anion (2.658 (5) and 2.690 (6) Å) while O(water)···O(water) distances are prolonged (2.746 (5) and 2.747 (5) Å, respectively). The building units are accommodated so that the O···O distances between water and cations are the longest (from 2.899 (4) to 2.963 (4) Å). The reason for significantly longer O···O aforementioned contacts are two pairs of bifurcated hydrogen bonds, donored by O1w and O3w. The details about hydrogen bonding can be seen in Table 1.

When considering the hydrogen bond topology, firstly, the sulfonamide groups link cations into pairs *via* two N–H···O hydrogen bonds around the pseudo inversion centre that leads to the formation of *R*_2_^2^(8) rings. The cationic pairs are spatially arranged one above the other, *i.e.* there are columns of the cationic pairs in the structure. In between four of such pairs, anions are stacked, forming a larger structural segment (*i.e.* an anion in between four cationic pairs, 'A+4C'). This building unit is held together by N–H···O hydrogen bonds in which the anionic O atoms O1 and O3 are acceptors of two H-bonds being donnored by two neighbouring cationic columns. The other two anionic O atoms, *i.e.* O2 and O4, are in charge for further connections of the aforementioned larger structural segment 'A+4C', each *via* two water molecules, *e.g.* by a sequence of O–H···O hydrogen bonds which link together a cationic pair from one unit with the anion of the neighbouring unit. The described connections are depicted in Fig. 3.

### Experimental

Tamsulosin adipate was prepared by mixing tamsulosin base and adipic (hexanedioic) acid in acetone at reflux temperature. The solution was cooled, concentrated and filtered. Obtained tamsulosin adipate was dried and dissolved in water at 25 °C to obtain a clear solution. The solution was left to stand at 25 °C for 7 days. Precipitated crystals of the title compound were separated from mother solution. The starting base was optically pure *R* enantiomer as well as the final product were optically active ([α]_Na_^20 °C^=-6°, conc. in methanol = 4 mg ml^-1^), which proves that the chirality was preserved during synthesis.

### Refinement

All H atoms were observed in a difference Fourier map. All H atoms bonded to carbon atoms were put at their idealized positions and treated as riding with C–H distances 0.98 (methyl), 0.97 (methylene) and 0.93 Å (aromatic H atoms). The methyl groups were allowed to rotate. The temperature parameters of the methyl H atoms were set to *U*_iso_(H) = 1.5 *U*_eq_(C) of the parent carbon atom, for all other H atoms they were set to *U*_iso_(H) = 1.2 *U*_eq_(C). H atoms from the water molecules were found in a difference Fourier map; their coordinates were fixed while their displacement parameters were constrained to be *U*_iso_(H) = 1.2 *U*_eq_(O). Hydrogen atoms bonded to N atoms were obtained from the difference electron density map. To additionally prove the correct assignment and positioning of such hydrogen atoms, their coordinates were allowed to change according to *SHELXL97*'s AFIX 4 command (*i.e.* such hydrogen were treated as riding with the changeable N–H distance while the N–H direction did not change). N–H distance was restrained to 0.95 (2) Å while displacement parameters were set to be *U*_iso_(H) = 1.2 *U*_eq_(N).

### Crystal data

**Table:**

2C_20_H_29_N_2_O_5_S^+^·C_6_H_8_O_4_^2^^−^·4H_2_O	*Z* = 1
*M**_r_* = 1035.21	*F*(000) = 554
Triclinic, *P*1	*D*_x_ = 1.301 Mg m^−^^3^
Hall symbol: P 1	Mo *K*α radiation, λ = 0.71073 Å
*a* = 10.4595 (2) Å	Cell parameters from 5866 reflections
*b* = 11.9020 (3) Å	θ = 2.6–27.5°
*c* = 12.6423 (3) Å	µ = 0.17 mm^−^^1^
α = 69.439 (1)°	*T* = 293 K
β = 70.466 (1)°	Prism, colourless
γ = 67.058 (1)°	0.25 × 0.25 × 0.20 mm
*V* = 1320.95 (5) Å^3^	

### Data collection

**Table:**

Nonius KappaCCD diffractometer	10988 independent reflections
Radiation source: fine-focus sealed tube	8651 reflections with *I* > 2σ(*I*)
Graphite monochromator	*R*_int_ = 0.030
φ and ω scans	θ_max_ = 27.5°, θ_min_ = 2.9°
Absorption correction: multi-scan (*DENZO-SMN*; Otwinowski & Minor, 1997)	*h* = −13→13
*T*_min_ = 0.933, *T*_max_ = 0.966	*k* = −15→15
26272 measured reflections	*l* = −16→16

### Refinement

**Table:**

Refinement on *F*^2^	Secondary atom site location: difference Fourier map
Least-squares matrix: full	Hydrogen site location: inferred from neighbouring sites
*R*[*F*^2^ > 2σ(*F*^2^)] = 0.047	H-atom parameters constrained
*wR*(*F*^2^) = 0.143	*w* = 1/[σ^2^(*F*_o_^2^) + (0.082*P*)^2^ + 0.3804*P*] where *P* = (*F*_o_^2^ + 2*F*_c_^2^)/3
*S* = 1.00	(Δ/σ)_max_ = 0.012
10988 reflections	Δρ_max_ = 0.42 e Å^−^^3^
641 parameters	Δρ_min_ = −0.36 e Å^−^^3^
7 restraints	Absolute structure: Flack (1983), 4978 Friedel pairs
Primary atom site location: structure-invariant direct methods	Flack parameter: 0.10 (8)

### Special details

**Table:**

Geometry. All e.s.d.'s (except the e.s.d. in the dihedral angle between two l.s. planes) are estimated using the full covariance matrix. The cell e.s.d.'s are taken into account individually in the estimation of e.s.d.'s in distances, angles and torsion angles; correlations between e.s.d.'s in cell parameters are only used when they are defined by crystal symmetry. An approximate (isotropic) treatment of cell e.s.d.'s is used for estimating e.s.d.'s involving l.s. planes.
Refinement. Refinement of *F*^2^ against ALL reflections. The weighted *R*-factor *wR* and goodness of fit *S* are based on *F*^2^, conventional *R*-factors *R* are based on *F*, with *F* set to zero for negative *F*^2^. The threshold expression of *F*^2^ > σ(*F*^2^) is used only for calculating *R*-factors(gt) *etc*. and is not relevant to the choice of reflections for refinement. *R*-factors based on *F*^2^ are statistically about twice as large as those based on *F*, and *R*- factors based on ALL data will be even larger.

### Fractional atomic coordinates and isotropic or equivalent isotropic displacement parameters (Å^2^)

**Table:**

	*x*	*y*	*z*	*U*_iso_*/*U*_eq_	
O1	0.6496 (4)	0.5164 (3)	0.0736 (3)	0.0565 (9)	
O2	0.7569 (5)	0.3443 (4)	0.0177 (3)	0.0905 (14)	
O3	0.1453 (3)	0.1548 (3)	0.4551 (3)	0.0523 (8)	
O4	0.0387 (5)	0.3273 (3)	0.5150 (3)	0.0777 (12)	
C1	0.6746 (4)	0.4005 (4)	0.0907 (4)	0.0442 (10)	
C2	0.5971 (5)	0.3303 (4)	0.2015 (4)	0.0477 (11)	
H2A	0.6176	0.3394	0.2669	0.057*	
H2B	0.6324	0.2413	0.2037	0.057*	
C3	0.4355 (5)	0.3774 (4)	0.2138 (3)	0.0450 (10)	
H3A	0.3981	0.4620	0.2248	0.054*	
H3B	0.4157	0.3820	0.1425	0.054*	
C4	0.3585 (4)	0.2935 (4)	0.3147 (3)	0.0421 (10)	
H4A	0.3953	0.2090	0.3034	0.050*	
H4B	0.3787	0.2884	0.3860	0.050*	
C5	0.1984 (5)	0.3409 (4)	0.3272 (4)	0.0462 (10)	
H5A	0.1770	0.3318	0.2622	0.055*	
H5B	0.1634	0.4299	0.3250	0.055*	
C6	0.1220 (4)	0.2694 (4)	0.4398 (4)	0.0415 (9)	
S1	0.18986 (9)	−0.00121 (8)	0.22314 (7)	0.0430 (2)	
N1	0.2480 (4)	−0.0319 (3)	0.3356 (3)	0.0467 (9)	
H1A	0.2095 (12)	0.037 (2)	0.3710 (11)	0.056*	
H1B	0.346 (3)	−0.0821 (16)	0.3247 (4)	0.056*	
N2	−0.1459 (3)	0.6004 (3)	−0.1238 (3)	0.0376 (7)	
H2C	−0.1640 (4)	0.6259 (4)	−0.1995 (10)	0.045*	
H2D	−0.2078 (9)	0.5522 (7)	−0.0727 (7)	0.045*	
O11	0.2622 (4)	−0.1080 (3)	0.1734 (3)	0.0539 (8)	
O12	0.0377 (3)	0.0360 (3)	0.2568 (3)	0.0631 (10)	
O13	0.4740 (3)	0.0202 (3)	0.1156 (3)	0.0476 (7)	
O14	−0.3472 (3)	0.8492 (2)	−0.2029 (2)	0.0416 (7)	
O15	−0.3762 (3)	0.9233 (3)	−0.4155 (2)	0.0443 (7)	
C11	0.2329 (4)	0.1324 (4)	0.1231 (3)	0.0357 (9)	
C12	0.3758 (4)	0.1302 (4)	0.0791 (3)	0.0369 (8)	
C13	0.4067 (5)	0.2386 (4)	0.0041 (4)	0.0474 (10)	
H13	0.5007	0.2395	−0.0248	0.057*	
C14	0.2944 (5)	0.3470 (4)	−0.0276 (4)	0.0497 (11)	
H14	0.3159	0.4177	−0.0815	0.060*	
C15	0.1554 (4)	0.3519 (4)	0.0180 (4)	0.0408 (9)	
C16	0.1232 (4)	0.2435 (3)	0.0934 (3)	0.0367 (8)	
H16	0.0285	0.2449	0.1239	0.044*	
C17	0.6170 (5)	0.0194 (5)	0.0968 (4)	0.0553 (12)	
H17A	0.6534	0.0513	0.0156	0.083*	
H17B	0.6757	−0.0654	0.1228	0.083*	
H17C	0.6179	0.0716	0.1395	0.083*	
C18	0.0310 (5)	0.4697 (3)	−0.0052 (3)	0.0428 (9)	
H18A	0.0449	0.5370	0.0118	0.051*	
H18B	−0.0551	0.4540	0.0487	0.051*	
C19	0.0067 (4)	0.5163 (3)	−0.1288 (3)	0.0418 (8)	
H19	0.0703	0.5665	−0.1784	0.050*	
C20	0.0324 (4)	0.4120 (3)	−0.1822 (3)	0.0577 (8)	
H20A	−0.0204	0.3559	−0.1297	0.086*	
H20B	0.1323	0.3660	−0.1971	0.086*	
H20C	0.0016	0.4476	−0.2538	0.086*	
C21	−0.1822 (5)	0.7154 (4)	−0.0838 (4)	0.0443 (10)	
H21A	−0.1192	0.7645	−0.1343	0.053*	
H21B	−0.1680	0.6906	−0.0058	0.053*	
C22	−0.3367 (5)	0.7961 (4)	−0.0847 (3)	0.0418 (9)	
H22A	−0.3998	0.7447	−0.0438	0.050*	
H22B	−0.3640	0.8626	−0.0465	0.050*	
C23	−0.4814 (4)	0.9261 (3)	−0.2209 (3)	0.0392 (9)	
C24	−0.5956 (5)	0.9652 (4)	−0.1320 (4)	0.0481 (10)	
H24	−0.5858	0.9385	−0.0558	0.058*	
C25	−0.7254 (5)	1.0458 (5)	−0.1603 (5)	0.0584 (12)	
H25	−0.8029	1.0727	−0.1021	0.070*	
C26	−0.7398 (5)	1.0853 (4)	−0.2721 (5)	0.0569 (13)	
H26	−0.8264	1.1399	−0.2895	0.068*	
C27	−0.6266 (5)	1.0447 (4)	−0.3602 (4)	0.0486 (11)	
H27	−0.6377	1.0706	−0.4359	0.058*	
C28	−0.4974 (4)	0.9656 (4)	−0.3347 (4)	0.0389 (9)	
C29	−0.3867 (5)	0.9648 (5)	−0.5339 (4)	0.0557 (12)	
H29A	−0.4197	1.0563	−0.5577	0.067*	
H29B	−0.4547	0.9337	−0.5424	0.067*	
C30	−0.2435 (6)	0.9161 (6)	−0.6079 (4)	0.0683 (15)	
H30A	−0.1738	0.9361	−0.5901	0.102*	
H30B	−0.2458	0.9545	−0.6882	0.102*	
H30C	−0.2187	0.8262	−0.5931	0.102*	
S2	0.60656 (9)	0.66650 (8)	0.31156 (7)	0.0439 (2)	
N11	0.5491 (4)	0.6979 (4)	0.1995 (3)	0.0544 (10)	
H11A	0.5659 (7)	0.647 (2)	0.1666 (13)	0.065*	
H11B	0.461 (3)	0.7428 (17)	0.2154 (7)	0.065*	
N12	0.9489 (4)	0.0739 (3)	0.6452 (3)	0.0428 (8)	
H12A	1.0225 (10)	0.1083 (6)	0.5916 (7)	0.051*	
H12B	0.9646 (4)	0.0530 (4)	0.7214 (10)	0.051*	
O21	0.5347 (4)	0.7730 (3)	0.3602 (3)	0.0536 (8)	
O22	0.7593 (4)	0.6269 (3)	0.2803 (3)	0.0700 (11)	
O23	0.3185 (3)	0.6474 (3)	0.4169 (3)	0.0517 (8)	
O24	1.1348 (3)	−0.1864 (3)	0.7396 (2)	0.0432 (7)	
O25	1.1648 (3)	−0.2583 (3)	0.9491 (2)	0.0453 (7)	
C31	0.5598 (4)	0.5330 (4)	0.4112 (3)	0.0400 (9)	
C32	0.4169 (4)	0.5365 (4)	0.4548 (4)	0.0424 (10)	
C33	0.3869 (5)	0.4294 (4)	0.5306 (5)	0.0538 (12)	
H33	0.2928	0.4312	0.5659	0.065*	
C34	0.4958 (5)	0.3202 (5)	0.5540 (4)	0.0548 (12)	
H34	0.4731	0.2474	0.6014	0.066*	
C35	0.6403 (5)	0.3133 (4)	0.5095 (4)	0.0493 (11)	
C36	0.6673 (5)	0.4240 (4)	0.4382 (4)	0.0496 (11)	
H36	0.7613	0.4243	0.4077	0.059*	
C37	0.1771 (5)	0.6470 (5)	0.4389 (5)	0.0672 (15)	
H37A	0.1776	0.5791	0.4143	0.101*	
H37B	0.1227	0.7258	0.3967	0.101*	
H37C	0.1348	0.6359	0.5204	0.101*	
C38	0.7597 (6)	0.1903 (4)	0.5347 (4)	0.0572 (12)	
H38A	0.8398	0.1878	0.4679	0.069*	
H38B	0.7265	0.1196	0.5488	0.069*	
C39	0.8075 (4)	0.1791 (4)	0.6409 (3)	0.0493 (9)	
H39	0.8245	0.2583	0.6299	0.059*	
C40	0.6991 (4)	0.1575 (5)	0.7543 (3)	0.0792 (12)	
H40A	0.6114	0.2253	0.7501	0.119*	
H40B	0.7347	0.1544	0.8164	0.119*	
H40C	0.6825	0.0791	0.7682	0.119*	
C41	0.9714 (5)	−0.0457 (4)	0.6204 (4)	0.0507 (11)	
H41A	0.9543	−0.0271	0.5443	0.061*	
H41B	0.9021	−0.0853	0.6769	0.061*	
C42	1.1161 (5)	−0.1357 (4)	0.6235 (4)	0.0511 (11)	
H42A	1.1306	−0.2037	0.5912	0.061*	
H42B	1.1864	−0.0930	0.5763	0.061*	
C43	1.2695 (4)	−0.2615 (4)	0.7532 (3)	0.0371 (9)	
C44	1.3831 (5)	−0.3016 (5)	0.6684 (4)	0.0568 (12)	
H44	1.3726	−0.2762	0.5925	0.068*	
C45	1.5136 (6)	−0.3792 (5)	0.6927 (5)	0.0672 (14)	
H45	1.5899	−0.4063	0.6339	0.081*	
C46	1.5293 (5)	−0.4158 (5)	0.8044 (5)	0.0631 (14)	
H46	1.6169	−0.4678	0.8213	0.076*	
C47	1.4161 (5)	−0.3761 (4)	0.8921 (4)	0.0490 (11)	
H47	1.4284	−0.4007	0.9673	0.059*	
C48	1.2845 (4)	−0.2999 (4)	0.8685 (3)	0.0366 (9)	
C49	1.1750 (6)	−0.2944 (5)	1.0684 (4)	0.0579 (12)	
H49A	1.2399	−0.2585	1.0751	0.069*	
H49B	1.2115	−0.3855	1.0947	0.069*	
C50	1.0326 (6)	−0.2480 (5)	1.1399 (4)	0.0661 (14)	
H50A	0.9915	−0.1599	1.1066	0.099*	
H50B	1.0400	−0.2598	1.2170	0.099*	
H50C	0.9732	−0.2939	1.1429	0.099*	
O1W	0.9470 (4)	−0.0136 (3)	0.8819 (3)	0.0628 (9)	
H1WA	1.0223	−0.0842	0.8889	0.075*	
H1WB	0.9329	0.0289	0.9381	0.075*	
O2W	0.9007 (4)	0.0962 (4)	0.0550 (4)	0.0800 (12)	
H2WA	0.8489	0.1829	0.0462	0.096*	
H2WB	0.9253	0.0837	0.1285	0.096*	
O3W	−0.1434 (3)	0.6917 (3)	−0.3570 (2)	0.0537 (8)	
H3WA	−0.2261	0.7583	−0.3650	0.064*	
H3WB	−0.1390	0.6378	−0.4008	0.064*	
O4W	0.8976 (5)	0.5726 (4)	0.4780 (4)	0.0810 (12)	
H4WA	0.9473	0.4914	0.4931	0.097*	
H4WB	0.8304	0.5939	0.4364	0.097*	

### Atomic displacement parameters (Å^2^)

**Table:**

	*U*^11^	*U*^22^	*U*^33^	*U*^12^	*U*^13^	*U*^23^
O1	0.071 (2)	0.0392 (17)	0.0538 (17)	−0.0240 (15)	−0.0103 (16)	−0.0007 (13)
O2	0.094 (3)	0.074 (2)	0.061 (2)	−0.009 (2)	0.022 (2)	−0.0241 (19)
O3	0.0575 (19)	0.0375 (16)	0.0513 (17)	−0.0196 (13)	0.0034 (14)	−0.0085 (13)
O4	0.092 (3)	0.0450 (18)	0.065 (2)	−0.0189 (18)	0.017 (2)	−0.0141 (16)
C1	0.040 (2)	0.041 (2)	0.043 (2)	−0.0135 (18)	−0.0062 (18)	−0.0028 (18)
C2	0.045 (2)	0.040 (2)	0.048 (2)	−0.0135 (18)	−0.0098 (19)	0.0000 (18)
C3	0.050 (2)	0.045 (2)	0.0354 (19)	−0.0199 (19)	−0.0083 (19)	−0.0006 (17)
C4	0.043 (2)	0.039 (2)	0.041 (2)	−0.0177 (17)	−0.0077 (18)	−0.0022 (16)
C5	0.049 (2)	0.043 (2)	0.042 (2)	−0.0194 (19)	−0.0081 (19)	−0.0012 (17)
C6	0.038 (2)	0.039 (2)	0.044 (2)	−0.0129 (17)	−0.0075 (18)	−0.0079 (17)
S1	0.0410 (5)	0.0356 (5)	0.0466 (5)	−0.0139 (4)	−0.0112 (4)	−0.0002 (4)
N1	0.060 (2)	0.0373 (18)	0.0337 (16)	−0.0069 (17)	−0.0119 (16)	−0.0062 (14)
N2	0.0378 (16)	0.0348 (16)	0.0356 (15)	−0.0108 (12)	−0.0094 (13)	−0.0033 (12)
O11	0.066 (2)	0.0427 (17)	0.0592 (19)	−0.0194 (15)	−0.0197 (16)	−0.0119 (14)
O12	0.0371 (16)	0.054 (2)	0.077 (2)	−0.0167 (14)	−0.0107 (16)	0.0087 (17)
O13	0.0329 (14)	0.0435 (16)	0.0583 (18)	−0.0065 (12)	−0.0152 (13)	−0.0050 (14)
O14	0.0394 (14)	0.0420 (15)	0.0323 (13)	−0.0041 (11)	−0.0127 (11)	−0.0020 (11)
O15	0.0473 (16)	0.0453 (16)	0.0314 (13)	−0.0067 (13)	−0.0115 (12)	−0.0056 (11)
C11	0.0317 (18)	0.036 (2)	0.040 (2)	−0.0109 (15)	−0.0142 (16)	−0.0031 (16)
C12	0.0328 (19)	0.036 (2)	0.042 (2)	−0.0086 (16)	−0.0108 (16)	−0.0096 (16)
C13	0.044 (2)	0.054 (3)	0.046 (2)	−0.020 (2)	−0.0089 (19)	−0.010 (2)
C14	0.053 (2)	0.038 (2)	0.052 (2)	−0.0177 (18)	−0.013 (2)	0.0005 (18)
C15	0.040 (2)	0.033 (2)	0.049 (2)	−0.0054 (16)	−0.0217 (17)	−0.0062 (16)
C16	0.0336 (18)	0.036 (2)	0.0388 (19)	−0.0081 (15)	−0.0153 (16)	−0.0036 (16)
C17	0.034 (2)	0.062 (3)	0.072 (3)	−0.010 (2)	−0.020 (2)	−0.017 (2)
C18	0.051 (2)	0.0293 (18)	0.0402 (19)	0.0022 (15)	−0.0186 (17)	−0.0080 (14)
C19	0.0359 (16)	0.0335 (16)	0.0447 (18)	−0.0057 (12)	−0.0111 (14)	−0.0008 (13)
C20	0.066 (2)	0.0532 (19)	0.0473 (17)	−0.0068 (15)	−0.0165 (15)	−0.0152 (14)
C21	0.053 (2)	0.032 (2)	0.049 (2)	−0.0128 (18)	−0.0212 (19)	−0.0034 (17)
C22	0.048 (2)	0.040 (2)	0.0321 (17)	−0.0098 (16)	−0.0143 (16)	−0.0025 (15)
C23	0.039 (2)	0.030 (2)	0.044 (2)	−0.0072 (16)	−0.0103 (18)	−0.0079 (16)
C24	0.048 (2)	0.051 (2)	0.042 (2)	−0.0109 (19)	−0.0050 (19)	−0.0169 (19)
C25	0.037 (2)	0.062 (3)	0.068 (3)	−0.001 (2)	−0.005 (2)	−0.029 (2)
C26	0.035 (2)	0.051 (3)	0.084 (3)	−0.0033 (19)	−0.025 (2)	−0.017 (2)
C27	0.049 (2)	0.044 (2)	0.057 (3)	−0.0086 (19)	−0.032 (2)	−0.0053 (19)
C28	0.039 (2)	0.033 (2)	0.045 (2)	−0.0127 (16)	−0.0081 (17)	−0.0096 (16)
C29	0.064 (3)	0.055 (3)	0.046 (2)	−0.013 (2)	−0.029 (2)	0.000 (2)
C30	0.076 (4)	0.094 (4)	0.035 (2)	−0.035 (3)	−0.012 (2)	−0.009 (2)
S2	0.0419 (5)	0.0370 (5)	0.0497 (5)	−0.0132 (4)	−0.0150 (5)	−0.0019 (4)
N11	0.054 (2)	0.048 (2)	0.048 (2)	−0.0023 (18)	−0.0148 (18)	−0.0086 (17)
N12	0.0408 (17)	0.0409 (18)	0.0382 (16)	−0.0153 (13)	−0.0134 (14)	0.0070 (13)
O21	0.070 (2)	0.0360 (16)	0.0600 (19)	−0.0175 (14)	−0.0262 (16)	−0.0055 (14)
O22	0.0437 (18)	0.062 (2)	0.089 (3)	−0.0227 (16)	−0.0130 (18)	0.0038 (19)
O23	0.0337 (15)	0.0454 (17)	0.068 (2)	−0.0036 (13)	−0.0168 (15)	−0.0101 (15)
O24	0.0474 (16)	0.0428 (15)	0.0312 (13)	−0.0080 (12)	−0.0127 (12)	−0.0033 (11)
O25	0.0465 (16)	0.0501 (17)	0.0330 (14)	−0.0111 (13)	−0.0126 (13)	−0.0042 (12)
C31	0.043 (2)	0.033 (2)	0.043 (2)	−0.0136 (17)	−0.0135 (18)	−0.0036 (17)
C32	0.042 (2)	0.039 (2)	0.049 (2)	−0.0100 (18)	−0.0181 (19)	−0.0093 (18)
C33	0.036 (2)	0.047 (3)	0.073 (3)	−0.018 (2)	−0.008 (2)	−0.009 (2)
C34	0.061 (3)	0.048 (3)	0.062 (3)	−0.030 (2)	−0.023 (2)	0.002 (2)
C35	0.061 (3)	0.044 (2)	0.046 (2)	−0.021 (2)	−0.018 (2)	−0.0040 (18)
C36	0.045 (2)	0.046 (2)	0.059 (3)	−0.018 (2)	−0.019 (2)	−0.004 (2)
C37	0.038 (2)	0.067 (3)	0.100 (4)	−0.004 (2)	−0.022 (3)	−0.031 (3)
C38	0.068 (3)	0.048 (3)	0.068 (3)	−0.025 (2)	−0.032 (2)	−0.005 (2)
C39	0.053 (2)	0.055 (2)	0.0369 (17)	−0.0219 (16)	−0.0107 (15)	−0.0023 (15)
C40	0.054 (2)	0.101 (3)	0.052 (2)	−0.009 (2)	−0.0073 (17)	−0.0058 (19)
C41	0.063 (3)	0.054 (3)	0.038 (2)	−0.023 (2)	−0.025 (2)	0.0040 (18)
C42	0.072 (3)	0.040 (2)	0.0343 (19)	−0.0095 (19)	−0.0176 (19)	−0.0043 (16)
C43	0.040 (2)	0.035 (2)	0.036 (2)	−0.0128 (16)	−0.0113 (17)	−0.0062 (15)
C44	0.056 (3)	0.056 (3)	0.052 (3)	−0.009 (2)	−0.012 (2)	−0.015 (2)
C45	0.052 (3)	0.074 (3)	0.071 (3)	−0.013 (3)	−0.006 (3)	−0.027 (3)
C46	0.046 (3)	0.064 (3)	0.077 (3)	−0.008 (2)	−0.018 (3)	−0.021 (3)
C47	0.049 (2)	0.048 (3)	0.053 (2)	−0.020 (2)	−0.016 (2)	−0.006 (2)
C48	0.043 (2)	0.033 (2)	0.0358 (19)	−0.0150 (16)	−0.0149 (16)	−0.0015 (15)
C49	0.067 (3)	0.080 (3)	0.034 (2)	−0.030 (3)	−0.010 (2)	−0.014 (2)
C50	0.075 (4)	0.075 (4)	0.038 (2)	−0.023 (3)	−0.006 (2)	−0.010 (2)
O1W	0.0567 (19)	0.063 (2)	0.0516 (17)	0.0036 (15)	−0.0139 (15)	−0.0181 (15)
O2W	0.081 (3)	0.067 (2)	0.108 (3)	0.0004 (19)	−0.042 (2)	−0.048 (2)
O3W	0.0537 (17)	0.0573 (17)	0.0391 (14)	0.0001 (14)	−0.0165 (13)	−0.0134 (12)
O4W	0.083 (3)	0.065 (2)	0.106 (3)	−0.001 (2)	−0.044 (2)	−0.039 (2)

### Geometric parameters (Å, º)

**Table:**

O1—C1	1.251 (5)	C29—H29B	0.9700
O2—C1	1.222 (5)	C30—H30A	0.9600
O3—C6	1.243 (5)	C30—H30B	0.9600
O4—C6	1.255 (5)	C30—H30C	0.9600
C1—C2	1.505 (5)	S2—O21	1.430 (3)
C2—C3	1.530 (6)	S2—O22	1.433 (3)
C2—H2A	0.9700	S2—N11	1.590 (4)
C2—H2B	0.9700	S2—C31	1.771 (4)
C3—C4	1.522 (3)	N11—H11A	0.7839
C3—H3A	0.9700	N11—H11B	0.8609
C3—H3B	0.9700	N12—C41	1.477 (6)
C4—C5	1.515 (6)	N12—C39	1.524 (6)
C4—H4A	0.9700	N12—H12A	0.9601
C4—H4B	0.9700	N12—H12B	0.9601
C5—C6	1.519 (5)	O23—C32	1.363 (5)
C5—H5A	0.9700	O23—C37	1.412 (6)
C5—H5B	0.9700	O24—C43	1.373 (5)
S1—O12	1.429 (3)	O24—C42	1.427 (5)
S1—O11	1.440 (3)	O25—C48	1.367 (5)
S1—N1	1.602 (4)	O25—C49	1.445 (5)
S1—C11	1.760 (4)	C31—C36	1.368 (6)
N1—H1A	0.9540	C31—C32	1.399 (6)
N1—H1B	0.9575	C32—C33	1.377 (6)
N2—C21	1.492 (5)	C33—C34	1.369 (7)
N2—C19	1.511 (5)	C33—H33	0.9300
N2—H2C	0.9564	C34—C35	1.406 (6)
N2—H2D	0.9564	C34—H34	0.9300
O13—C12	1.351 (5)	C35—C36	1.383 (6)
O13—C17	1.431 (5)	C35—C38	1.523 (7)
O14—C23	1.388 (5)	C36—H36	0.9300
O14—C22	1.427 (4)	C37—H37A	0.9600
O15—C28	1.379 (5)	C37—H37B	0.9600
O15—C29	1.430 (5)	C37—H37C	0.9600
C11—C16	1.401 (5)	C38—C39	1.531 (6)
C11—C12	1.403 (5)	C38—H38A	0.9700
C12—C13	1.389 (5)	C38—H38B	0.9700
C13—C14	1.405 (6)	C39—C40	1.512 (5)
C13—H13	0.9300	C39—H39	0.9800
C14—C15	1.359 (6)	C40—H40A	0.9600
C14—H14	0.9300	C40—H40B	0.9600
C15—C16	1.396 (5)	C40—H40C	0.9600
C15—C18	1.513 (5)	C41—C42	1.478 (7)
C16—H16	0.9300	C41—H41A	0.9700
C17—H17A	0.9600	C41—H41B	0.9700
C17—H17B	0.9600	C42—H42A	0.9700
C17—H17C	0.9600	C42—H42B	0.9700
C18—C19	1.537 (5)	C43—C44	1.362 (6)
C18—H18A	0.9700	C43—C48	1.410 (5)
C18—H18B	0.9700	C44—C45	1.381 (8)
C19—C20	1.509 (5)	C44—H44	0.9300
C19—H19	0.9800	C45—C46	1.371 (8)
C20—H20A	0.9600	C45—H45	0.9300
C20—H20B	0.9600	C46—C47	1.381 (7)
C20—H20C	0.9600	C46—H46	0.9300
C21—C22	1.526 (6)	C47—C48	1.385 (6)
C21—H21A	0.9700	C47—H47	0.9300
C21—H21B	0.9700	C49—C50	1.466 (7)
C22—H22A	0.9700	C49—H49A	0.9700
C22—H22B	0.9700	C49—H49B	0.9700
C23—C24	1.394 (6)	C50—H50A	0.9600
C23—C28	1.394 (6)	C50—H50B	0.9600
C24—C25	1.400 (7)	C50—H50C	0.9600
C24—H24	0.9300	O1W—H1WA	0.9011
C25—C26	1.364 (7)	O1W—H1WB	0.9531
C25—H25	0.9300	O2W—H2WA	0.9461
C26—C27	1.388 (7)	O2W—H2WB	0.9924
C26—H26	0.9300	O3W—H3WA	0.9248
C27—C28	1.379 (6)	O3W—H3WB	0.9642
C27—H27	0.9300	O4W—H4WA	0.8873
C29—C30	1.491 (7)	O4W—H4WB	0.9218
C29—H29A	0.9700		
			
O2—C1—O1	121.4 (4)	C27—C28—C23	120.0 (4)
O2—C1—C2	119.5 (4)	O15—C29—C30	108.9 (4)
O1—C1—C2	119.1 (4)	O15—C29—H29A	109.9
C1—C2—C3	112.8 (3)	C30—C29—H29A	109.9
C1—C2—H2A	109.0	O15—C29—H29B	109.9
C3—C2—H2A	109.0	C30—C29—H29B	109.9
C1—C2—H2B	109.0	H29A—C29—H29B	108.3
C3—C2—H2B	109.0	C29—C30—H30A	109.5
H2A—C2—H2B	107.8	C29—C30—H30B	109.5
C4—C3—C2	113.2 (2)	H30A—C30—H30B	109.5
C4—C3—H3A	108.9	C29—C30—H30C	109.5
C2—C3—H3A	108.9	H30A—C30—H30C	109.5
C4—C3—H3B	108.9	H30B—C30—H30C	109.5
C2—C3—H3B	108.9	O21—S2—O22	118.1 (2)
H3A—C3—H3B	107.8	O21—S2—N11	107.1 (2)
C5—C4—C3	113.0 (2)	O22—S2—N11	108.6 (2)
C5—C4—H4A	109.0	O21—S2—C31	109.55 (19)
C3—C4—H4A	109.0	O22—S2—C31	105.57 (19)
C5—C4—H4B	109.0	N11—S2—C31	107.5 (2)
C3—C4—H4B	109.0	S2—N11—H11A	122.6
H4A—C4—H4B	107.8	S2—N11—H11B	105.4
C4—C5—C6	111.9 (3)	H11A—N11—H11B	116.1
C4—C5—H5A	109.2	C41—N12—C39	120.4 (3)
C6—C5—H5A	109.2	C41—N12—H12A	107.3
C4—C5—H5B	109.2	C39—N12—H12A	107.2
C6—C5—H5B	109.2	C41—N12—H12B	107.2
H5A—C5—H5B	107.9	C39—N12—H12B	107.3
O3—C6—O4	122.0 (4)	H12A—N12—H12B	106.8
O3—C6—C5	118.8 (4)	C32—O23—C37	117.7 (4)
O4—C6—C5	119.1 (4)	C43—O24—C42	116.5 (3)
O12—S1—O11	117.8 (2)	C48—O25—C49	118.2 (4)
O12—S1—N1	107.7 (2)	C36—C31—C32	120.8 (4)
O11—S1—N1	107.7 (2)	C36—C31—S2	118.2 (3)
O12—S1—C11	105.73 (17)	C32—C31—S2	120.8 (3)
O11—S1—C11	109.37 (18)	O23—C32—C33	125.5 (4)
N1—S1—C11	108.19 (18)	O23—C32—C31	116.2 (4)
S1—N1—H1A	112.9	C33—C32—C31	118.3 (4)
S1—N1—H1B	110.6	C34—C33—C32	119.9 (4)
H1A—N1—H1B	123.5	C34—C33—H33	120.0
C21—N2—C19	114.6 (3)	C32—C33—H33	120.0
C21—N2—H2C	108.7	C33—C34—C35	122.8 (4)
C19—N2—H2C	108.6	C33—C34—H34	118.6
C21—N2—H2D	108.6	C35—C34—H34	118.6
C19—N2—H2D	108.6	C36—C35—C34	115.9 (4)
H2C—N2—H2D	107.6	C36—C35—C38	122.1 (4)
C12—O13—C17	118.7 (3)	C34—C35—C38	121.9 (4)
C23—O14—C22	116.0 (3)	C31—C36—C35	122.0 (4)
C28—O15—C29	117.1 (3)	C31—C36—H36	119.0
C16—C11—C12	120.2 (3)	C35—C36—H36	119.0
C16—C11—S1	119.5 (3)	O23—C37—H37A	109.5
C12—C11—S1	120.2 (3)	O23—C37—H37B	109.5
O13—C12—C13	124.9 (4)	H37A—C37—H37B	109.5
O13—C12—C11	116.0 (3)	O23—C37—H37C	109.5
C13—C12—C11	119.1 (4)	H37A—C37—H37C	109.5
C12—C13—C14	119.3 (4)	H37B—C37—H37C	109.5
C12—C13—H13	120.4	C35—C38—C39	110.2 (4)
C14—C13—H13	120.4	C35—C38—H38A	109.6
C15—C14—C13	122.1 (4)	C39—C38—H38A	109.6
C15—C14—H14	118.9	C35—C38—H38B	109.6
C13—C14—H14	118.9	C39—C38—H38B	109.6
C14—C15—C16	119.0 (4)	H38A—C38—H38B	108.1
C14—C15—C18	124.0 (4)	C40—C39—N12	110.8 (3)
C16—C15—C18	117.0 (4)	C40—C39—C38	114.0 (4)
C15—C16—C11	120.2 (4)	N12—C39—C38	108.4 (4)
C15—C16—H16	119.9	C40—C39—H39	107.8
C11—C16—H16	119.9	N12—C39—H39	107.8
O13—C17—H17A	109.5	C38—C39—H39	107.8
O13—C17—H17B	109.5	C39—C40—H40A	109.5
H17A—C17—H17B	109.5	C39—C40—H40B	109.5
O13—C17—H17C	109.5	H40A—C40—H40B	109.5
H17A—C17—H17C	109.5	C39—C40—H40C	109.5
H17B—C17—H17C	109.5	H40A—C40—H40C	109.5
C15—C18—C19	116.2 (3)	H40B—C40—H40C	109.5
C15—C18—H18A	108.2	N12—C41—C42	113.5 (4)
C19—C18—H18A	108.2	N12—C41—H41A	108.9
C15—C18—H18B	108.2	C42—C41—H41A	108.9
C19—C18—H18B	108.2	N12—C41—H41B	108.9
H18A—C18—H18B	107.4	C42—C41—H41B	108.9
N2—C19—C20	108.8 (3)	H41A—C41—H41B	107.7
N2—C19—C18	107.8 (3)	O24—C42—C41	110.8 (4)
C20—C19—C18	114.0 (3)	O24—C42—H42A	109.5
N2—C19—H19	108.7	C41—C42—H42A	109.5
C20—C19—H19	108.7	O24—C42—H42B	109.5
C18—C19—H19	108.7	C41—C42—H42B	109.5
C19—C20—H20A	109.5	H42A—C42—H42B	108.1
C19—C20—H20B	109.5	O24—C43—C44	126.5 (4)
H20A—C20—H20B	109.5	O24—C43—C48	113.9 (3)
C19—C20—H20C	109.5	C44—C43—C48	119.6 (4)
H20A—C20—H20C	109.5	C45—C44—C43	121.4 (5)
H20B—C20—H20C	109.5	C45—C44—H44	119.3
N2—C21—C22	110.9 (4)	C43—C44—H44	119.3
N2—C21—H21A	109.5	C44—C45—C46	119.3 (5)
C22—C21—H21A	109.5	C44—C45—H45	120.4
N2—C21—H21B	109.5	C46—C45—H45	120.4
C22—C21—H21B	109.5	C45—C46—C47	120.5 (5)
H21A—C21—H21B	108.0	C45—C46—H46	119.7
O14—C22—C21	107.7 (3)	C47—C46—H46	119.7
O14—C22—H22A	110.2	C48—C47—C46	120.4 (5)
C21—C22—H22A	110.2	C48—C47—H47	119.8
O14—C22—H22B	110.2	C46—C47—H47	119.8
C21—C22—H22B	110.2	O25—C48—C47	125.2 (4)
H22A—C22—H22B	108.5	O25—C48—C43	116.1 (4)
C24—C23—C28	120.5 (4)	C47—C48—C43	118.7 (4)
C24—C23—O14	123.0 (4)	O25—C49—C50	108.6 (4)
C28—C23—O14	116.5 (3)	O25—C49—H49A	110.0
C23—C24—C25	118.3 (4)	C50—C49—H49A	110.0
C23—C24—H24	120.9	O25—C49—H49B	110.0
C25—C24—H24	120.9	C50—C49—H49B	110.0
C26—C25—C24	120.9 (4)	H49A—C49—H49B	108.3
C26—C25—H25	119.5	C49—C50—H50A	109.5
C24—C25—H25	119.5	C49—C50—H50B	109.5
C25—C26—C27	120.7 (4)	H50A—C50—H50B	109.5
C25—C26—H26	119.7	C49—C50—H50C	109.5
C27—C26—H26	119.7	H50A—C50—H50C	109.5
C26—C27—C28	119.6 (5)	H50B—C50—H50C	109.5
C26—C27—H27	120.2	H1WA—O1W—H1WB	109.4
C28—C27—H27	120.2	H2WA—O2W—H2WB	95.9
O15—C28—C27	124.7 (4)	H3WA—O3W—H3WB	105.2
O15—C28—C23	115.2 (4)	H4WA—O4W—H4WB	114.1
			
O2—C1—C2—C3	−115.3 (5)	C24—C23—C28—C27	0.4 (6)
O1—C1—C2—C3	62.3 (6)	O14—C23—C28—C27	−178.5 (4)
C1—C2—C3—C4	170.3 (3)	C28—O15—C29—C30	176.5 (4)
C2—C3—C4—C5	179.6 (5)	O21—S2—C31—C36	−128.8 (4)
C3—C4—C5—C6	−170.0 (3)	O22—S2—C31—C36	−0.7 (5)
C4—C5—C6—O3	−63.9 (5)	N11—S2—C31—C36	115.1 (4)
C4—C5—C6—O4	115.3 (5)	O21—S2—C31—C32	55.6 (4)
O12—S1—C11—C16	−0.6 (4)	O22—S2—C31—C32	−176.3 (4)
O11—S1—C11—C16	127.2 (4)	N11—S2—C31—C32	−60.5 (4)
N1—S1—C11—C16	−115.8 (3)	C37—O23—C32—C33	−12.6 (7)
O12—S1—C11—C12	175.4 (4)	C37—O23—C32—C31	166.9 (4)
O11—S1—C11—C12	−56.8 (4)	C36—C31—C32—O23	−176.0 (4)
N1—S1—C11—C12	60.2 (4)	S2—C31—C32—O23	−0.5 (6)
C17—O13—C12—C13	13.7 (6)	C36—C31—C32—C33	3.5 (7)
C17—O13—C12—C11	−165.5 (4)	S2—C31—C32—C33	179.0 (4)
C16—C11—C12—O13	177.9 (4)	O23—C32—C33—C34	174.1 (5)
S1—C11—C12—O13	1.9 (5)	C31—C32—C33—C34	−5.4 (8)
C16—C11—C12—C13	−1.3 (6)	C32—C33—C34—C35	4.2 (9)
S1—C11—C12—C13	−177.3 (3)	C33—C34—C35—C36	−1.0 (8)
O13—C12—C13—C14	179.7 (4)	C33—C34—C35—C38	−178.7 (5)
C11—C12—C13—C14	−1.2 (7)	C32—C31—C36—C35	−0.3 (7)
C12—C13—C14—C15	3.9 (7)	S2—C31—C36—C35	−176.0 (4)
C13—C14—C15—C16	−3.9 (7)	C34—C35—C36—C31	−1.0 (7)
C13—C14—C15—C18	174.3 (4)	C38—C35—C36—C31	176.7 (5)
C14—C15—C16—C11	1.3 (6)	C36—C35—C38—C39	89.2 (6)
C18—C15—C16—C11	−177.1 (4)	C34—C35—C38—C39	−93.3 (5)
C12—C11—C16—C15	1.3 (6)	C41—N12—C39—C40	83.6 (5)
S1—C11—C16—C15	177.3 (3)	C41—N12—C39—C38	−42.3 (5)
C14—C15—C18—C19	69.6 (6)	C35—C38—C39—C40	70.1 (5)
C16—C15—C18—C19	−112.1 (4)	C35—C38—C39—N12	−165.9 (3)
C21—N2—C19—C20	−174.1 (3)	C39—N12—C41—C42	177.6 (3)
C21—N2—C19—C18	61.7 (4)	C43—O24—C42—C41	−173.6 (4)
C15—C18—C19—N2	159.5 (3)	N12—C41—C42—O24	71.0 (5)
C15—C18—C19—C20	38.6 (5)	C42—O24—C43—C44	−6.5 (6)
C19—N2—C21—C22	178.4 (3)	C42—O24—C43—C48	174.9 (4)
C23—O14—C22—C21	179.9 (3)	O24—C43—C44—C45	−178.8 (5)
N2—C21—C22—O14	−69.4 (4)	C48—C43—C44—C45	−0.2 (7)
C22—O14—C23—C24	9.0 (6)	C43—C44—C45—C46	−0.5 (9)
C22—O14—C23—C28	−172.1 (4)	C44—C45—C46—C47	0.2 (8)
C28—C23—C24—C25	−0.6 (7)	C45—C46—C47—C48	0.8 (8)
O14—C23—C24—C25	178.2 (4)	C49—O25—C48—C47	0.8 (6)
C23—C24—C25—C26	−0.2 (8)	C49—O25—C48—C43	−179.5 (4)
C24—C25—C26—C27	1.1 (8)	C46—C47—C48—O25	178.1 (4)
C25—C26—C27—C28	−1.3 (7)	C46—C47—C48—C43	−1.5 (7)
C29—O15—C28—C27	−0.2 (6)	O24—C43—C48—O25	0.3 (5)
C29—O15—C28—C23	−178.1 (4)	C44—C43—C48—O25	−178.5 (4)
C26—C27—C28—O15	−177.3 (4)	O24—C43—C48—C47	180.0 (4)
C26—C27—C28—C23	0.5 (7)	C44—C43—C48—C47	1.2 (6)
C24—C23—C28—O15	178.5 (4)	C48—O25—C49—C50	−175.5 (4)
O14—C23—C28—O15	−0.4 (5)		

### Hydrogen-bond geometry (Å, º)

**Table:**

*D*—H···*A*	*D*—H	H···*A*	*D*···*A*	*D*—H···*A*
N1—H1*B*···O21^i^	0.96	2.11	3.028 (5)	160
N11—H11*B*···O11^ii^	0.86	2.23	3.029 (5)	155
N2—H2*C*···O3*W*	0.96	1.84	2.755 (4)	160
N12—H12*B*···O1*W*	0.96	1.87	2.797 (4)	160
N2—H2*D*···O1^iii^	0.96	1.98	2.854 (4)	151
N12—H12*A*···O3^iv^	0.96	1.83	2.746 (4)	159
N1—H1*A*···O3	0.95	1.84	2.790 (5)	173
N11—H11*A*···O1	0.78	2.03	2.798 (6)	169
O1*W*—H1*WB*···O2*W*^v^	0.95	1.80	2.746 (5)	174
O3*W*—H3*WB*···O4*W*^vi^	0.96	1.82	2.747 (5)	161
O2*W*—H2*WA*···O2	0.95	1.75	2.690 (6)	175
O4*W*—H4*WA*···O4^iv^	0.89	1.77	2.658 (5)	175
O1*W*—H1*WA*···O24	0.90	2.33	2.899 (4)	121
O1*W*—H1*WA*···O25	0.90	2.08	2.963 (4)	165
O3*W*—H3*WA*···O14	0.92	2.39	2.920 (4)	116
O3*W*—H3*WA*···O15	0.92	2.03	2.935 (4)	166

Symmetry codes: (i) *x*, *y*−1, *z*; (ii) *x*, *y*+1, *z*; (iii) *x*−1, *y*, *z*; (iv) *x*+1, *y*, *z*; (v) *x*, *y*, *z*+1; (vi) *x*−1, *y*, *z*−1.
